# Artificial intelligence processing electronic health records to identify commonalities and comorbidities cluster at Immuno Center Humanitas

**DOI:** 10.1002/clt2.12144

**Published:** 2022-06-08

**Authors:** Pierandrea Morandini, Maria Elena Laino, Giovanni Paoletti, Alessandro Carlucci, Tobia Tommasini, Giovanni Angelotti, Jack Pepys, Giorgio Walter Canonica, Enrico Heffler, Victor Savevski, Francesca Puggioni

**Affiliations:** ^1^ Artificial Intelligence Center IRCCS Humanitas Research Hospital Milan Italy; ^2^ Department of Biomedical Sciences Humanitas University Milan Italy; ^3^ Personalized Medicine, Asthma and Allergy IRCCS Humanitas Research Hospital Milan Italy

**Keywords:** allergy, artificial intelligence, asthma, clustering, natural language processing, urticaria

## Abstract

**Background:**

Comorbidities are common in chronic inflammatory conditions, requiring multidisciplinary treatment approach. Understanding the link between a single disease and its comorbidities is important for appropriate treatment and management. We evaluate the ability of an NLP‐based process for knowledge discovery to detect information about pathologies, patients' phenotype, doctors' prescriptions and commonalities in electronic medical records, by extracting information from free narrative text written by clinicians during medical visits, resulting in the extraction of valuable information and enriching real world evidence data from a multidisciplinary setting.

**Methods:**

We collected clinical notes from the Allergy Department of Humanitas Research Hospital written in the last 3 years and used it to look for diseases that cluster together as comorbidities associated to the main pathology of our patients, and for the extent of prescription of systemic corticosteroids, thus evaluating the ability of NLP‐based tools for knowledge discovery to extract structured information from free text.

**Results:**

We found that the 3 most frequent comorbidities to appear in our clusters were asthma, rhinitis, and urticaria, and that 991 (of 2057) patients suffered from at least one of these comorbidities. The clusters which co‐occur particularly often are oral allergy syndrome and urticaria (131 patients), angioedema and urticaria (105 patients), rhinitis and asthma (227 patients). With regards to systemic corticosteroid prescription volume by our clinicians, we found it was lower when compared to the therapy the patients followed before coming to our attention, with the exception of two diseases: Chronic obstructive pulmonary disease and Angioedema.

**Conclusions:**

This analysis seems to be valid and is confirmed by the data from the literature. This means that NLP tools could have significant role in many other research fields of medicine, as it may help identify other important, and possibly previously neglected clusters of patients with comorbidities and commonalities. Another potential benefit of this approach lies in its potential ability to foster a multidisciplinary approach, using the same drugs to treat pathologies normally treated by physicians in different branches of medicine, thus saving resources and improving the pharmacological management of patients.

## 1 INTRODUCTION

Comorbidity is common in autoimmune or inflammatory conditions, such as asthma,^1^ chronic obstructive pulmonary disease (COPD),^2^ rheumatoid arthritis,^3^ psoriasis and psoriatic arthritis,^4^ and inflammatory bowel disease (IBD)^5^ with 30% of patients manifesting more than one condition and thus requiring a multidisciplinary approach.^6^

Assessing how and when comorbidities are associated with a major condition would provide a deeper understanding of the comorbidity itself and, at the same time, provide new insights for a better treatment strategy.

Based on this background and taking advantage of data warehouse (DWH) resources of the Humanitas Immuno Center, our aim is to evaluate the ability of NLP‐based tools for knowledge discovery to detect information about pathologies in medical records collected from free text format. Medical records are written by clinical professionals in a narrative style during hospital visits. As a main outcome, we expect to use patients' data to identify the different pathologies treated in our Allergy Department, understand if there are any comorbidity associations, and extract positive feedback for the practical management of these patients.

Indeed, this would allow more precise patients' phenotyping and tailored therapies, reducing both active and passive costs related to poor control of the disease, and improving the quality of life of the patients.^7^, ^8^, ^9^

## 2 MATERIALS AND METHODS

### 2.1 Dataset

We retrospectively collected all the clinical notes written from January 2017 to September 2020 of patients with ongoing or terminated care process at the Allergy Department of Humanitas Research Hospital.

We included in our study only medical records from patients who gave their consent for the use of their data for research purposes.

We excluded the hospital records collected during encounters with only therapeutic purposes (i.e., visits for drug infusion), since these records do not contain relevant information for our analysis.

### 2.2 Data selection

The clinical notes we processed present multiple layout structures, hence the information we collected is generally located in different paragraphs of the clinical notes.

In this regard, a normalization of the clinical notes was required in order to standardize the data for the downstream processes. An analysis of the used layouts led to the identification of the paragraphs containing the information we are interested in.

In particular, the only paragraphs we considered in our analysis were those related to the patient's *anamnesis*, in which the searched pathologies are considered as comorbidities and drugs are considered as previous therapy, the *conclusions* paragraph to extract the final diagnosis, and the *therapy* paragraph to extract the drugs prescribed by our clinicians.

This method of data analysis was selected following consultations with the allergy unit clinicians on their standardized method of reporting.

The complete list of considered comorbidities can be found in Table 1 and the list of systemic corticosteroids can be found in Table 2.

**TABLE 1:** List of comorbidities reported in the anamnesis paragraph

List of comorbidities	
Anafilassi	Anaphylaxis
Angioedema	Angioedema
Arterite	Arteritis
Artrite psoriasica	Psoriatic arthritis
Artrite reumatoide	Rheumatoid arthritis
Asma	Asthma
Aspergillosi	Aspergillosis
Bronchiectasie	Bronchiectasis
Broncopneumopatia cronica ostruttiva (BPCO)	Chronic Obstructive Pulmonary Disease (COPD)
Churg strauss	Churg strauss
Colite indeterminata	Indeterminate colitis
Colite ulcerosa	Ulcerative colitis
Connettivite	Connectivitis
Dattilite	Dactylitis
Dermatite atopica	Atopic dermatitis
Esofagite eosinofila	Eosinophilic esophagitis
Interstiziopatia	Interstitial disease
Lupus	Lupus
Mastocitosi	Mastocytosis
Miosite	Myositis
Morbo di Crohn	Crohn's disease
Orticaria	Urticaria
Osteoporosi	Osteoporosis
Poliposi nasale	Nasal polyposis
Polmonite eosinofila	Eosinophilic pneumonia
Psoriasi	Psoriasis
Rinite	Rhinitis
Rinosinusite	Rhinosinusitis
Sacroileite	Sacroiliitis
Sclerosi sistemica	Systemic sclerosis
Sindrome orale allergica (SOA)	Oral allergic syndrome (SOA)
Sinusite	Sinusitis
Sjogren	Sjogren
Spondilite	Spondylitis
Spondiloartrite	Spondyloarthritis
Vasculiti	Vasculitis

**TABLE 2:** Patients treated with a specific systemic corticosteroid drug

Corticosteroid drugs	Prescription	Anamnesis
Cortisone	2	31
Prednisone	131	152
Prednisolone	0	0
Methylprednisolone	9	30
Beclomethasone	5	22
Triamcinolone	2	15
Budesonide	13	20
Betamethasone	66	59
Dexamethasone	0	2
Hydrocortisone	1	13
Not specified corticosteroid	0	203

*Note*: In column “Prescription”, we included the number of patients prescribed with a specific or not specified corticosteroid in our center. In the column “Anamnesis”, we included the number of patients previously treated with a specific or not specified corticosteroid.

The selections of pathologies and drugs were carried out in consultation with ImmunoCenter experts and literature analysis. Finally, the list of the diseases (36) and drugs (10 active principles and 31 tradenames) was identified focusing on those treated/prescribed through the multidisciplinary approach within the Humanitas ImmunoCenter.

### 2.3 Data pre‐processing

The first data extraction step consisted in querying the data from DWH. We used Oracle SQL^TM^ to gather the relevant data of patients examined at the Allergy Department. Consequently, a pre‐process pipeline was implemented to clean the text data from unwanted or unnecessary characters, returning a cleaned corpus ready to be processed. The pre‐processing phase aimed to both normalize the characters to ASCII format, and remove all HTML special characters from the text.

### 2.4 Marker extraction

For the whole of the following analysis, we used Python (ver. 3.6.9), including multiple libraries: *pandas* ^10^ *kmodes* ^11^ *regex* (*re*)^12^ and *scikit‐learn* ^13^ among others.

The marker extraction was performed entirely with Regular Expressions (RegEx), described in detail in Supporting information S1. The marker extraction process helped define reliable patterns used to detect the presence of the considered pathologies and therapies in the retrieved text. From this point onwards, we refer to those pathologies as ***entities***.

### 2.5 Evaluation of marker extraction process

We sampled a subset of sentences to manually evaluate the goodness of the extracted markers. For each pathology, we validated the extracted marker of 20 sentences. The first 10 sentences were presumed to express the presence of the pathology, 6 were supposed to express negations of pathology, and 4 were control sentences in which the pathology was not detected. We evaluated a binary outcome depending on the presence or absence of the disease. This allowed us to evaluate our algorithm with the indexes of Recall, Precision and F1 score.

### 2.6 Clustering

The dataset underwent a clustering process to explore optimal grouping arrangements of the gathered entities. The aim is to find the main families of clinical conditions considering, for each patient, both comorbidities and diagnosis.

All markers related to each hospital encounter were aggregated, resulting in a list of all the different autoimmune pathologies present along the care process for every patient.

Since the used data are composed only by binary flags, the clustering was performed with the k‐modes algorithm.^14^ This is a variation of the well‐known k‐means algorithm^15^ specifically fitted to work with binary data.

To define the optimal number of clusters traditional methods, base the clustering evaluation on metrics regarding the spatial distances between observations and their cluster centroids. Since it is not possible to define a spatial distance between categorical data, we relied on the cost function defined by the k‐modes algorithm, to find the optimal number of clusters.

The cost function is defined as:

$$P\left(W,\;Q\right)=\overset{k}{\underset{l=1}{\sum }}\overset{n}{\underset{i=1}{\sum }}\overset{m}{\underset{j=1}{\sum }}w_{i,l}\delta \left(x_{i,j},q_{l,j}\right)\;\left(1\right)$$

where

$$\delta \left(x_{j},\;y_{j}\right)=\left\{\begin{array}{l} 0\;\left(x_{j}=y_{j}\right)\\ 1\;\left(x_{j}\neq y_{j}\right) \end{array}\right.$$

Subject to

$$\overset{k}{\underset{l=1}{\sum }}w_{i,l}=1,\;1\leq i\leq n$$

$$w_{i,l}\in \left\{0,1\right\},\;1\leq i\leq n,\;1\leq l\leq k$$

These equations define the cost function as the sum of dissimilarities between a data point X, composed of m categorical attributes and n observations, and a matrix $Q=\left[q_{1},q_{2},\text{⋯},q_{k}\right]$ defining the modes of k clusters. These dissimilarities are weighted by the coefficients of a matrix W.

As suggested by Huang et al.^14^ to solve the Equation (1) an iterative process is needed. In particular, the values of W and Q are found by following these steps:

1. Fix $\hat{Q}=Q_{t}$ and solve $P\left(W,\hat{Q}\right)$ to obtain $W_{t}$
2. Fix $\hat{W}=W_{t}$ and solve $P\left(\hat{W},Q\right)$ to obtain $Q_{t+1}$

## 3 RESULTS

### 3.1 Marker extraction evaluation performance

To validate the performances of regular expression a total of 720 sentences were manually annotated. The values for recall, precision and F1 score were respectively 0.97, 0.84, and 0.90.

### 3.2 Hospital encounters analysis

In our Allergy Department, 3162 patients had 7827 visits from January 2017 to September 2020. Of these, we enrolled 2057 patients [887 (43.1%) men with the median age of 48.07 years (+20.59 s.d.)] after screening for the type of hospital visit and excluding patients who came only for therapeutic purposes. In this way, the number of visits was reduced to 3226 (average of 1.57 visits per patient).

**FIGURE 1:**
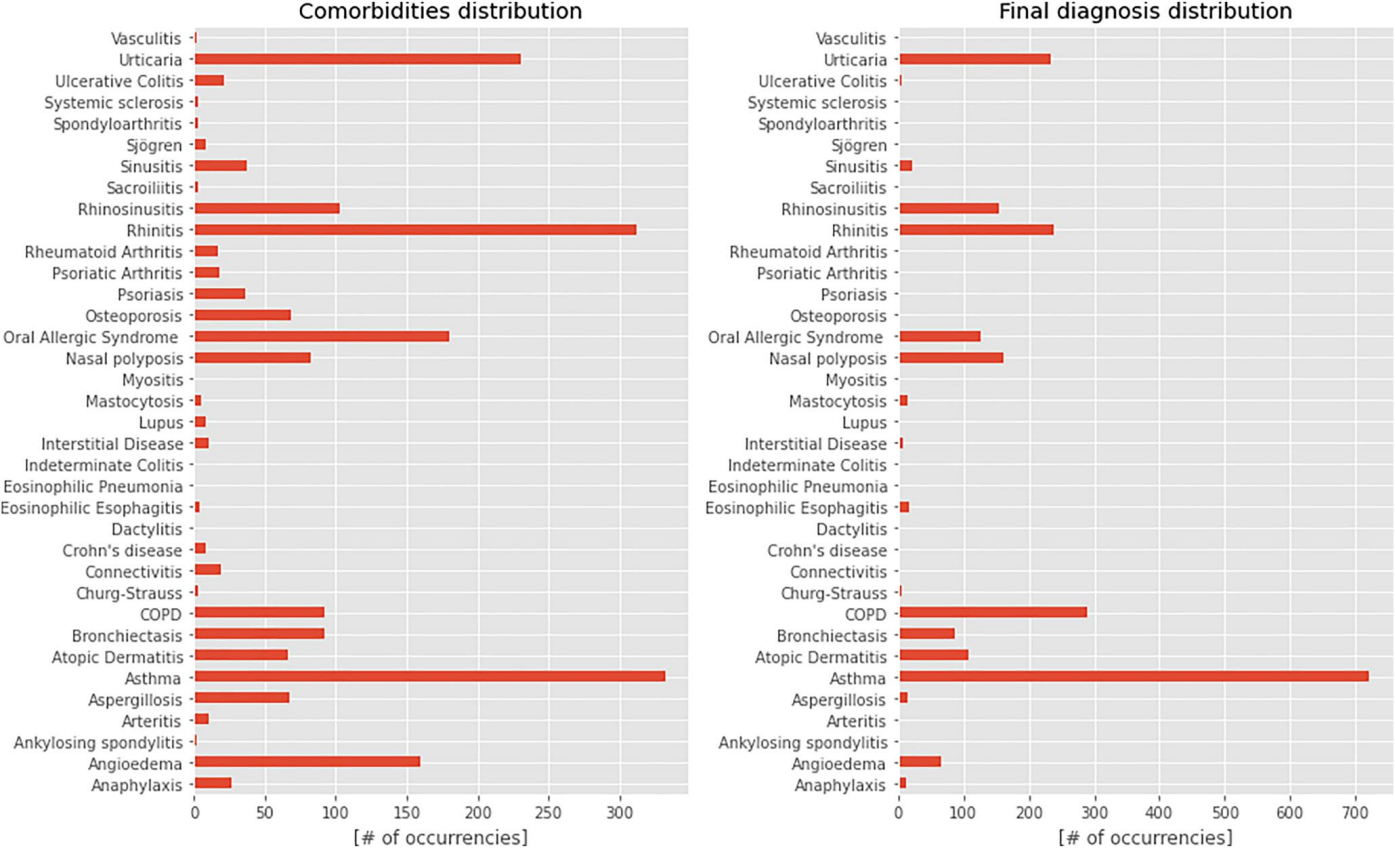
Distribution of diseases after the marker extraction considering a single hospital encounter. The first plot shows all the pathologies contained in the paragraph “anamnesis”—which we considered as comorbidities—and the second plot shows all the pathologies contained in the paragraph “conclusions”—which we considered as final diagnosis after the hospital encounter. The most commonly occurring comorbidities were asthma (detected in 332 hospital encounters, 10.2% of considered visits), rhinitis (312, 9.6%), and urticaria (230, 7.1%). Asthma is the most frequent pathology diagnosed (719 hospital encounters, 22.2%), followed by COPD (290, 8.9%) and rhinitis (239, 7.4%)

Figure 1 shows the distribution of diseases after the marker extraction step, before the aggregation to the patient level. In particular, it indicates how many clinical notes reported the pathologies of interest during a single hospital encounter.

As previously mentioned, we considered the diseases reported in the paragraphs “anamnesis” as comorbidities (left side of the figure) and those cited in the paragraph “conclusions” as diagnosis (right side of the figure).

In our series, the three most frequent comorbidities are *asthma* (detected in 332 hospital encounters, 10.2% of considered visits), *rhinitis* (312, 9.6%) and *urticaria* (230, 7.1%).

Moreover, *asthma* is the most frequent pathology diagnosed (719 hospital encounters, 22.2%), followed by *COPD* (90, 8.9%) and *rhinitis* (239, 7.4%).

**FIGURE 2:**
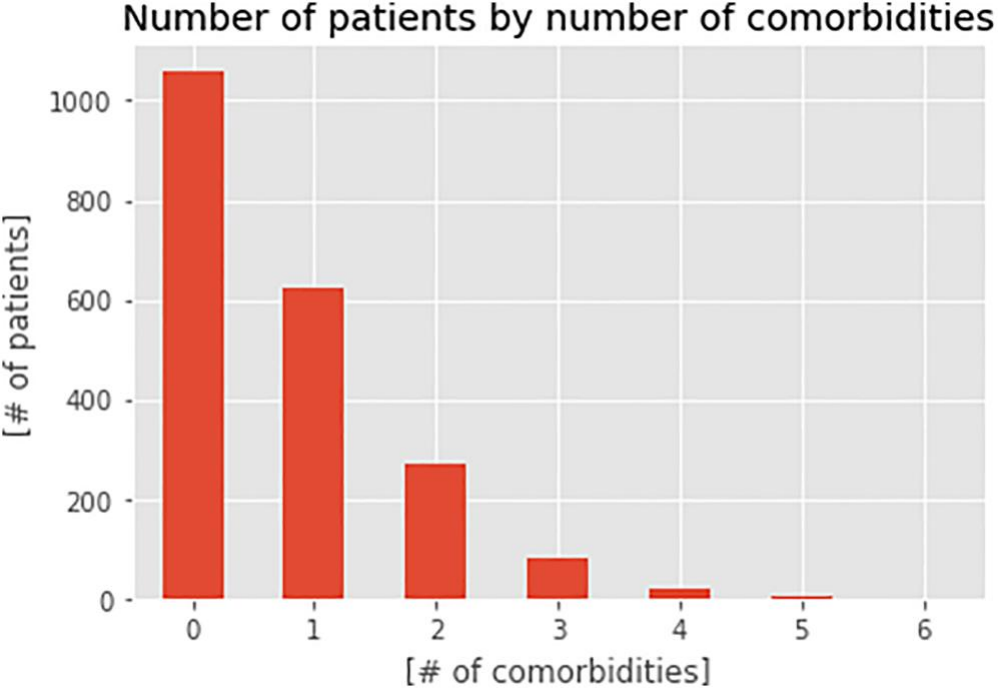
Volume of patients with comorbidities. This bar plot shows the volumes of patients as a function of the number of comorbidities found in the paragraph “anamnesis.” In particular, in our series 1066 did not show any comorbidities, 629 showed 1 comorbidity, 260 showed 2 comorbidities, 77 showed 3 comorbidities, 20 showed 4 comorbidities, 4 showed 5 comorbidities, 1 showed 6 comorbidities

After data aggregation and selection steps described in the section “Clustering”, we analyzed how many comorbidities were reported in clinical notes for each patient, considering each hospital encounter, as shown in Figure 2. We found that 991 out of 2057 patients suffered from at least one of the considered comorbidities, for a total of 1465 over 3226 hospital encounters, while 1066 patients were considered as not presenting the searched pathologies as we did not find the relevant comorbidities in their anamnesis paragraph.

**FIGURE 3:**
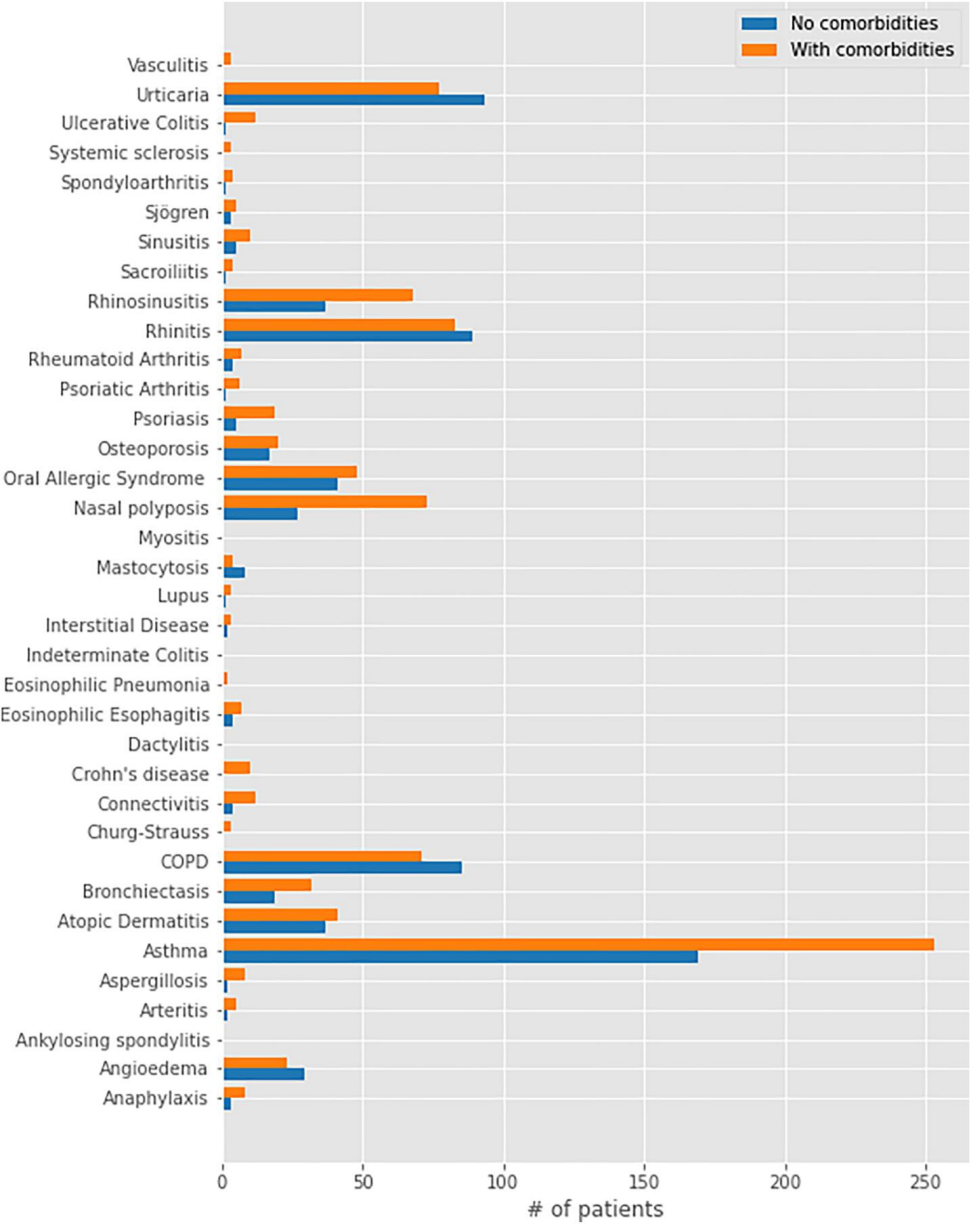
Distribution of the final diagnosis between patients with at least one comorbidity and patients with no comorbidity. We compared the raw counting of the distribution in the two groups of patients since they are composed of a very similar number of observations (1066 without comorbidities vs. 991 with comorbidities). Between the two groups, the most important differences in volume of found markers between the two groups were for nasal polyps (73 in the first group and 27 in the second group), rhinosinusitis (68 vs. 37), bronchiectasis (32 vs. 19), and asthma (253 vs. 169)

Furthermore, we investigated differences or similarities between the two categories of patients (with or without comorbidities reported in the *anamnesis* paragraph). For this purpose, we compared the distribution of pathologies found in the *conclusion* paragraph between the two categories of patients, as shown in Figure 3.

In particular, we noticed differences in the volume of found markers for Nasal *Polyps*, *Rhinosinusitis*, *Bronchiectasis* and *Asthma*. The latter remains the most diagnosed pathology in both categories but clinical notes which did not contain the searched comorbidities closely follows.

In Table 3, we show the *p*‐values for the correlation between the presence of at least one comorbidity and the diagnosis of asthma, rhinosinusitis, polyps, or bronchiectasis. The correlation is statistically significant with the diagnosis of all of them. This could be explained by the fact that patients with these four diseases frequently have a comorbidity as widely demonstrated in the literature.^16^

**TABLE 3:** Statistical correlation between the indicated disease and the presence of at least one comorbidity, expressed as *p*‐value calculated using the Pearson's χ^2^ test

Disease	*p*‐Value
Asthma	<0.001
Rhinosinusitis	<0.001
Polyps	<0.001
Bronchiectasis	0.049

## 4 PATIENTS ANALYSIS

From this point onwards, the analysis was conducted with focus on the whole patients' cure process, instead of considering single hospital encounters.

**FIGURE 4:**
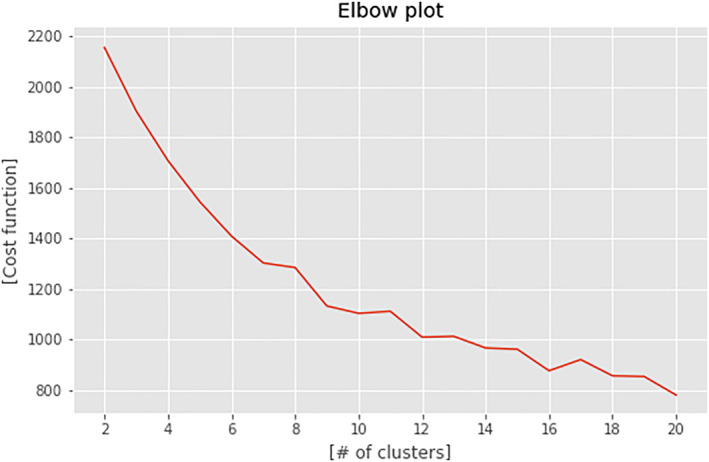
Clustering cost measure curve. Using the elbow method, *N* = 6 was the optimal number of clusters chosen in this setup

In order to define the optimal number of clusters to use in our study, we analyzed the elbow plot (Figure 4), which suggests that *N* = 6 is the best option for our series.

To confirm the goodness of clustering with *N* = 6, we analyzed the silhouette for each observation included in the clustering process.

**FIGURE 5:**
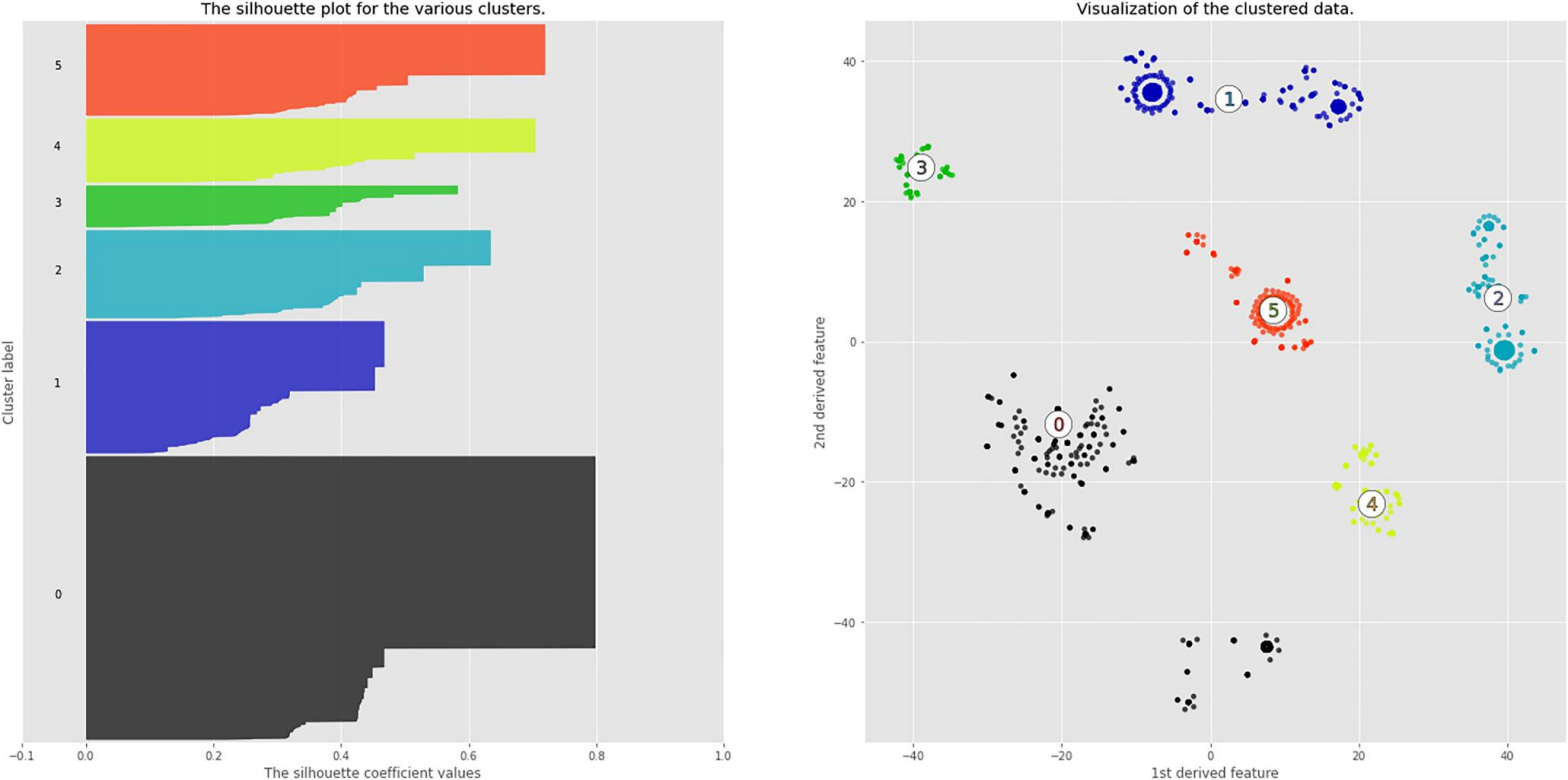
Patients' clusters in a fictitious 2D space. This figure represents the plots of the clustered patients in a fictitious 2D space (right side) and a plot in which the silhouette measure is calculated for each patient and plotted as part of the belonging cluster's silhouette. The silhouette measure indicates the goodness of clustering for each observation and its value ranges in the interval [–1, +1]. The larger the measure, the better the observation clustering. Based on this measure, all the different clusters have good ratings

As can be seen in Figure 5, for *N* = 6 all the observations have a silhouette relatively close to 1. We then characterized the different clusters in terms of comorbidities presence and numerosity in order to understand by which comorbidities they are defined.

**FIGURE 6:**
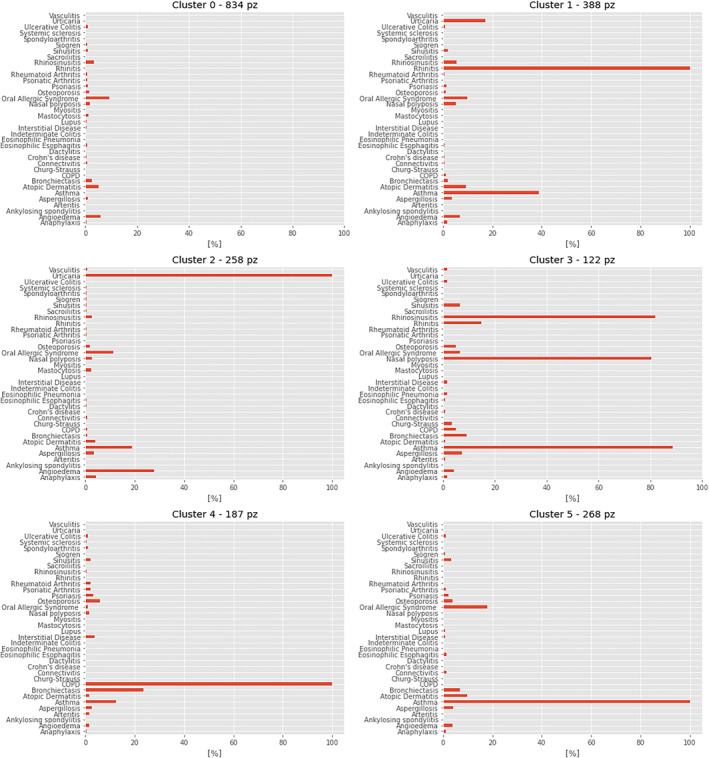
Cluster characterization by comorbidities. Each plot shows a specific cluster of patients in which we divided our series. The clusters have a different numerosity, as can be seen in the title of each plot. Furthermore, in each plot the percentage of patients with a specific comorbidity is represented

Figure 6 shows, for each cluster, the importance that the specific comorbidities have in characterizing the clusters (ratio between the cluster population and the number of those patients who experienced a certain comorbidity).

1. out of 6 clusters (clusters 1–5) showed a strong recurrence of a specific pathology.

Furthermore, in the above‐mentioned clusters, at least one secondary comorbidity seems to be correlated to the main one.

Specifically, *rhinitis* and *asthma* co‐occur in cluster 1 (388 patients), *angioedema* and *urticaria* in cluster 2 (258 patients), *asthma, rhinosinusitis and polyps* in cluster 3 (122 patients), *chronic obstructive pulmonary disease (COPD) and bronchiectasis in cluster 4 (187 patients), asthma and oral allergy syndrome in cluster 5 (268 patients).*

As can be seen, cluster 0 is not as defined as the others: no comorbidity is present in the majority of the population.

In cluster 0, the most common pathologies are *oral allergy syndrome*, *angioedema and atopic dermatitis.*

Furthermore, we analyzed how corticosteroid therapy correlated with assigned clusters. We collected all the information about prescription of systemic corticosteroids both in the anamnesis paragraph—which represented the therapy that patients followed before coming to our centre‐ and in the conclusive therapy paragraph—which included the drugs prescribed by our clinicians.

Analyzing the volumes of drugs prescribed in Humanitas and taken by patients before visits to our center, two differences are noticeable. The first one is that the volumes of drugs prescribed by our clinicians is lower than the volume reported in patients' anamnesis prior to treatment in our ImmunoCenter, as shown in Table 2. The reduction of prescriptions of these drugs is, indeed, an advantage of our center. Secondly, we found a difference between the patients treated with corticosteroids prior to visiting our clinicians and the patients to whom corticosteroids were prescribed by our clinicians—as shown in Table 4.

**TABLE 4:** Measures of overlap between newly prescribed and previous use of corticosteroids expressed as the number of patients who were prescribed corticosteroid along their care process

Corticosteroid drug	Before only	After only	Both
Cortisone	31	2	0
Prednisone	145	124	7
Prednisolone	0	0	0
Methylprednisolone	30	9	0
Beclomethasone	22	5	0
Triamcinolone	15	2	0
Budesonide	20	13	0
Betamethasone	58	65	1
Dexamethasone	2	0	0
Hydrocortisone	13	1	0
Not specified corticosteroid	189	215	14

*Note*: The column “Before only” contains the number of patients who were reported to use corticosteroids in the anamnesis and not in the prescriptions, the “After only” column contains the number of patients who were prescribed corticosteroids at our center, and in “Both” are the numbers of patients who were reported to use corticosteroids both before and after visiting the ImmunoCenter. In the last row “Not specified corticosteroid” we considered the previous use of a not specified corticosteroid (which means that there was no mention of the commercial name or of the active substance) and all the prescriptions made by our center.

Moreover, after analysing the correlation between drugs prescribed in our centre and patients' clusters, we found a significant correlation between prednisone and cluster 2 and betamethasone and clusters 2 and 4. As shown in Table 5, prednisone and betamethasone were the drugs that there was less of a reduction, or in the case of betamethasone, an increase in the prescription by our clinicians.

**TABLE 5:** *p*‐values indicating statistical correlation between patients' clusters (rows 0–5) and systemic corticosteroid

	Patients' clusters											
Corticosteroid drugs	0		1		2		3		4		5	
	Prescription	Anamnesis	Prescription	Anamnesis	Prescription	Anamnesis	Prescription	Anamnesis	Prescription	Anamnesis	Prescription	Anamnesis
Cortisone	0.654	0.980	0.824	0.278	0.595	0.832	0.253	0.612	0.434	0.841	0.615	0.432
Prednisone	0.007	0.479	0.229	0.542	0.000	0.157	0.071	0.863	0.898	0.432	0.135	0.500
Prednisolone	NaN	NaN	NaN	NaN	NaN	NaN	NaN	NaN	NaN	NaN	NaN	NaN
Methylprednisolone	0.208	0.804	0.306	0.586	0.708	0.335	0.962	0.180	0.712	0.884	0.745	0.823
Beclomethasone	0.631	0.855	0.612	0.722	0.864	0.867	0.700	0.859	0.000	1.000	0.840	0.687
Triamcinolone	0.654	0.759	0.824	0.827	0.595	0.765	0.253	0.669	0.434	0.902	0.615	0.050
Budesonide	0.662	0.858	0.973	0.321	0.913	0.501	0.750	0.514	0.509	0.303	0.135	0.944
Betamethasone	0.850	0.133	0.987	0.900	0.002	0.247	0.454	1.000	0.017	0.950	0.435	0.391
Dexamethasone	NaN	0.654	NaN	0.042321	NaN	0.594596	NaN	0.253342	NaN	0.433624	NaN	0.614832
Hydrocortisone	0.847	0.897	0.426	0.165	0.258	0.465	0.062	0.750	0.155	0.509	0.272	0.505
Not specified corticosteroid	NaN	0.549	NaN	0.405	NaN	0.450	NaN	0.742	NaN	0.667	NaN	0.959

*Note*: Under the semi‐columns “Prescription”, we included the *p*‐values related to the drugs prescribed in our center and each cluster; under the semi‐columns “Anamnesis”, we included the *p*‐values related to the drugs reported in the anamnesis paragraph (which were previously prescribed) and each cluster.

On the other side, there is no correlation between drugs found in the anamnesis and patients' clusters, as shown in Table 5.

## 5 DISCUSSION

We built a framework to extract structured information from free text through NLP, which can eventually be transposed to other types of clinical notes to extract valuable information to enrich real world evidence data.

After establishing the patients' inclusion criteria and the pathologies of interest for the study, we queried the data from the hospital's DWH. Subsequently, a pre‐processing pipeline was implemented in order to clean the text data from unwanted or unnecessary characters. Finally, the clinical notes underwent the marker extraction step, which consisted in the detection of the pathologies of interest (entities) in the analyzed clinical notes. The marker extraction process was carried out with regular expressions. This approach proved to be an efficient tool for the entity detection task on medical texts in an initially unsupervised fashion.

As stated by Wang,^17^ in medicine the information extraction tasks are mainly left to techniques that make use of empirical rules (as per regex) to obtain the requested results. One of the reasons justifying this preference is that rule‐based information extraction can incorporate domain knowledge from knowledge bases or experts, which is essential for clinical applications. In our study, we chose to use Regular Expressions instead of building an Entity Recognition model, which is a very time‐consuming option,^18^ mostly because of the high volume of annotated data required. Moreover, the task is suitable for RegEx approach because searched expressions of the considered pathologies are well defined as specific nouns due to the specificity of the medical terminology and presence of abbreviations. For these reasons, the action that may produce an alteration of the expressions can be caused only by misspelling or typing errors which are eventualities that could be handled by RegEx.

To test this hypothesis in our series, we evaluated our performances on a subset of sentences and obtained very good results. The high recall, in particular, can be explained by the method we used to validate. Sampling the sentences to annotate stratifying on the extracted marker is crucial to get a balanced set, but might introduce a bias. A more interesting parameter is the precision, which is still good, but not as good as the recall: this is caused by missed negations that precede or follow the mentioned pathology. This means that generally, it is possible to extract entities from clinical notes using RegEx being aware that it is crucial to focus also on the negations detection. With these data, it is possible to say that the marker extraction algorithm has acceptable performance, although a more in‐depth evaluation is required to better evaluate the performances of our algorithm.

Of note in our results is the fact that through regular expressions we retrieved epidemiologic data about our Allergy Department patients' phenotype.

We found that asthma is the pathology most frequently diagnosed. This data is due to different factors, as asthma affects up to 18% of the population^19^ and Humanitas' Allergy Unit is a world‐renowned center of excellence for asthma management and has performed several international clinical trials on asthma and comorbidities this is to be expected.

Similarly, Chronic rhinosinusitis with nasal polyps (CRSwNP) affects 5%–12% of the general population^20^ and is the second most frequent pathology managed by the Humanitas Allergy Unit, which is unsurprising, as it is often associated with severe asthma. The disease management in a multidisciplinary rhinology clinic by allergists and ENTs is another explanation for the frequency in which we encounter it.

Furthermore, as Figure 1 shows, in the first plot there is a more homogeneous distribution of the pathologies we took into consideration compared to the second plot. We expected this kind of distribution since we assumed that the anamnesis paragraph contained the information about comorbidities and the conclusions paragraph contained those about the diagnosis. This means that the second plot shows all the diseases treated in our Allergy Department, while in the first plot we can find diseases not directly treated in the Allergy Department but rather generally treated in our ImmunoCenter.

Retrieving information about the most frequent comorbidities in our series helped us get a more complete picture of our patients, which is very important in a multidisciplinary context.

Moreover, we found that four pathologies—asthma, rhinosinusitis, polyps, and bronchiectasis (Figure 3)—occur more frequently within the group of patients with at least one comorbidity, as a result of what is shown in Figure 3 and Table 3. This could suggest that the above‐mentioned diseases are more frequently associated with other diseases. This can be explained by the fact that asthma and rhinosinusitis with or without polyps can be driven by a common molecular mechanism, namely type 2 inflammation. This inflammatory response is emerging as a unifying feature of classically defined allergic diseases, such as asthma, and a range of other inflammatory diseases, such as rhinosinusitis^21^

For the other diseases, we did not find any substantial differences in the distribution of the final *diagnosis* between the group of patients with at least one comorbidity and the group with no comorbidities (Figure 3).

Interestingly, we found associations between different comorbidities, as shown in Figure 6. Specifically, in our clusters we found a co‐occurrence of:

· *Rhinitis* and *Asthma* (cluster 1),
· *Angioedema* and *Urticaria* (cluster 2)
· *Asthma, Rhinosinusitis,* and *Polyps (*cluster 3)
· *COPD* and *Bronchiectasis* (cluster 4)
· *Asthma* and *Oral Allergy Syndrome* (cluster 5)

When analysing these associations from a medical and pathophysiological point of view it is unsurprising to find them in the same patients, since they have the same endotype.

- Allergic rhinitis and asthma (cluster 1) are common diseases frequently occurring together. This association is known as “united airway disease.” Epidemiological studies have shown that the majority of patients with asthma have concomitant rhinitis and the presence of rhinitis is an increased risk factor for development of asthma^22^, ^23^, ^24^, ^25^, ^26^
- The underlying mechanism of the second cluster is mast cell degranulation, they are the primary effectors in urticaria and in many cases of angioedema,^27^ resulting in skin‐limited manifestations in urticaria while affecting the deeper layers in angioedema. It is therefore unsurprising that approximately 40% of patients with urticaria experience angioedema (cluster 2)^28^
- Patients with chronic rhinosinusitis with nasal polyps (CRSwNP) characterized by a type 2 immune inflammation often have severe and recurrent symptoms. Lower airway conditions such as asthma are common comorbidities and share similar pathophysiology (Cluster 3). CRSwNP with asthma is characterized by tissue eosinophilia and high local IgE levels. These conditions are correlated with more severe sinonasal symptoms and worse quality of life and clinical outcomes control^29^
- The prevalence of bronchiectasis in patients with COPD is high (cluster 4), especially in advanced stages. Some of the etiological factors for bronchiectasis are also present in patients with COPD and may be responsible for its development. Similarly, presence of a chronic bronchitis phenotype determines recurrent infective exacerbations, which perpetuate chronic inflammation, and tissue destruction^30^
- *Oral allergy syndrome* is a hypersensitivity reaction to plant‐based foods, manifesting most commonly with pruritus of the lips, tongue, and mouth. Unlike simple food allergy, this disease requires prior sensitization to a cross‐reacting inhalant allergen rather than direct sensitization to a specific food protein. However, a proportion of patients with oral allergic syndrome sensitized to certain pollens may have asthma (cluster 5) as an additional co‐morbidity^31^

One of the most interesting aspects, which we shall investigate in future research, is the correlation between pathology, treatment, clinical personal response to therapy and modification of the therapeutic approach in our multidisciplinary ImmunoCenter compared to what happens in a simple allergy unit. Results show that the corticosteroid prescription volume from our clinicians is lower compared to the therapy that patients followed prior to coming to our attention, except for two diseases: COPD and Angioedema.

There was a significant correlation between the prescription of prednisone by our clinicians and cluster 2 and betamethasone and clusters 2 and 4 (see Table 5). The explanation is that these drugs are recommended through an action plan as rescue medication in case of the appearance of severe angioedema^32^ or during severe exacerbation of COPD.^33^

### 5.1 Limitations of the study

A limitation of our study might be that most physicians have the tendency to focus on the pathologies of interest of their department. Thus, even assuming a correct extraction of the markers, we cannot exclude the omission of information relevant to the global health status of the patient.

Another limitation of the study can be the fact that we selected the pathologies of interest (comorbidities and diagnosis) before the marker extraction step. Therefore, the data we obtained may overlook useful information on the global health of a patient.

Furthermore, since we started from the analysis of free text, bias related to errors in sentence formatting (i.e., lack of punctuation) or spelling errors which may have influenced the marker extraction process cannot be excluded, even if the use of regular expressions aims at limiting this occurrence.

## 6 CONCLUSIONS

Regular expressions were proven as an effective tool for entity recognition to extract medical information from free text data and to retrieve epidemiological data in our ImmunoCenter and Allergy Department.

This analysis seems to be valid and is confirmed by data from the literature. This could have significant implications for many other clusters of patients in other fields of medicine, as it may help identify other important, and possibly previously neglected clusters, but above all to be able to identify new unknown clusters of patients affected by immune system's diseases.

Another potential benefit of this approach lies in its potential ability to save resources and improve pharmacological management of patients by using the same drugs^34^, ^35^, ^36^, ^37^, ^38^ to treat pathologies normally treated by physicians in different branches of medicine.

AI‐based methods of processing electronic medical records can contribute, as we have shown, to the creation of a new patient journey based on real word evidence Data Driven approach.

## CONFLICT OF INTEREST

Francesca Puggioni received reimbursements for lectures, presentations, speakers bureaus, manuscript writing or educational events from AstraZeneca, Mundipharma, Menarini, Almirall, Chiesi, Valeas, Malesci Guidotti, Boehringer Ingelheim, Sanofi, GSK, Novartis, Stallergenes‐Greer; for Consulting fees from Sanofi, Novartis, Stallergenes‐Greer. Giovanni Paoletti received reimbursements for lectures, presentations, speakers bureaus, manuscript writing or educational events from Lusopharma and Novartis. Enrico Heffler received reimbursements for lectures, presentations, speakers bureaus, manuscript writing or educational events from AstraZeneca, Sanofi, GSK, Novartis, Circassia, Nestlè Purina, Stallergenes‐Greer; for Consulting fees from AstraZeneca, Sanofi, GSK, Novartis, Circassia, Nestlè Purina, Stallergenes‐Greer. Giorgio Walter Canonica received reimbursements for lectures, presentations, speakers bureaus, manuscript writing or educational events from AstraZeneca, Sanofi, GSK, Novartis, Chiesi Farmaceutici, Hal Allergy, Menarini, Stallergenes‐Greer; for Consulting fees from AstraZeneca, Sanofi, GSK, Novartis, Chiesi Farmaceutici, Hal Allergy, Menarini, Stallergenes‐Greer; for Participation on a Data Safety Monitoring Board or Advisory Board from AstraZeneca, Sanofi, GSK, Novartis, Chiesi Farmaceutici, Hal Allergy, Menarini, Stallergenes‐Greer. The other authors declare that they have no conflict of interest to disclose regarding the publication of this manuscript.

## Supporting information

Supporting Information S1
