# Supplementary material for: Artificial intelligence processing electronic health records to identify commonalities and comorbidities cluster at Immuno Center Humanitas
Source: Clin Transl Allergy. 2022 Jun 8;12(6):e12144. doi: 10.1002/clt2.12144 (PMC9175261; doi:10.1002/clt2.12144)
Supplement: Supplementary file 1 — Supporting Information S1 [file CLT2-12-e12144-s001.doc]

**Supplemental Text**

**4.1 Entity Extraction**

For each entity a regular expression was defined. A regular expression is an elastic search pattern able to generalize the form of the text portion of interest. This allows for the retrieval of matches even with the presence of minor spelling errors, resulting in a solid way of locating the patterns of interest.

The following is an example of the regular expression used to detect the citation of nasal polyp and the relative matches are reported:

***RegEx:****(polip\w+\s+nas\w+)*

Matches:

•poliposi nasale (nasal polyposis)

•polipi nasali (nasal polyps)

•polipectomia nasale (nasal polypectomy)

•poliposi nasali (nasal polyposis)

•polipo nasale (nasal polyp)

**4.2 Negations**

Since we were looking for the presence of some specific comorbidities in the clinical state of a patient, it is mandatory to detect possible negations associated with the citation of a searched entity. Negations were detected using regular expressions that looked for words usually used in negations that were proposed for the relevant entity.

In the following example, we show the entity extraction result for negations in *nasal polyp* searching process:

•non poliposi nasale (no nasal polyposis)

•non recidiva di poliposi nasale (no recurrence of nasal polyposis)

•non ha evidenziato poliposi nasale (no evidence of nasal polyposis)

•non noti polipi nasali (no known nasal polyps)

•non segnala poliposi nasale (no nasal polyposis reported)

The finding of a negation preceding the relevant entity was considered as absence of the comorbidity.

The presence of words that suggest uncertainty about the presence of any pathologies was also considered as a negation.
